# Effects of Different Duration of High-Fat Feeding on the NAD^+^/Sirtuins/PGC1α Axis in Relation to Oxidative Stress in Rat Skeletal Muscle

**DOI:** 10.33549/physiolres.935706

**Published:** 2026-06-01

**Authors:** Xiujuan LIU, Yawen ZHENG, Xin ZHANG, Wanyu FENG

**Affiliations:** 1 Department of Sports and Health, Nanjing Sports Institute, Nanjing, China; 2 Department Science Experiment Center, Nanjing Sports Institute, Nanjing, China

**Keywords:** High-fat diet, Skeletal muscle, Oxidative stress, Obesity, NAD^+^, PGC1-α

## Abstract

This study examined the effects of varying durations of high-fat diet (HFD) exposure on oxidative stress and the NAD^+^/Sirtuins/PGC1α signaling pathway in rat skeletal muscle. Thirty-two male Sprague-Dawley rats were randomly assigned to either a control group or HFD groups with exposure periods of 4, 8, or 12 weeks. Outcome measures included body weight, inflammatory markers (IL-6, TNF-α, MCP-1), oxidative stress parameters (MDA, SOD, GSH), apoptotic activity, and protein expression levels of key components within the signaling pathway. The results demonstrated that body weight and systemic inflammation progressively increased with the duration of HFD intake. Markers of oxidative stress became significantly elevated by week 8 and further deteriorated by week 12, characterized by increased malondialdehyde (MDA) levels, diminished antioxidant capacity (reduced SOD and GSH), and enhanced apoptosis. A reduction in Sirt3 expression and increased acetylation of PGC1α were detected at 8 weeks, while by 12 weeks, dysregulation extended across the entire NAD^+^/Sirt1/PGC1α pathway. These alterations were associated with exacerbated oxidative damage. The findings suggest that the duration of HFD exposure plays a critical role in the development of skeletal muscle oxidative stress through progressive and time-dependent impairment of the NAD^+^/Sirtuins/PGC1α pathway.

## Introduction

In recent years, the global prevalence of overweight and obesity has been steadily rising. According to the World Obesity Federation’s 2025 World Obesity Report, Obesity has emerged as a truly global issue [1]. By 2030, it is projected that over 2.9 billion adults worldwide will be classified as obese [1]. Furthermore, health problems associated with obesity are also expected to worsen [2]. Currently, obesity is primarily attributed to high-calorie diets, particularly those rich in sugar or fat [3]. In recent years, there has been an increasing body of research highlighting the detrimental effects of high-sugar diets on human health [4–7], drawing significant attention. With evolving lifestyle patterns, the consumption of high-fat diets is likely to increase. Moreover, studies have demonstrated that high-fat diets can lead to distinct and severe consequences compared to high-sugar diets [3,8]. A high-fat diet (HFD) is defined as one where lipids account for more than 30 % of total energy intake [9]. Research indicates that high-fat diet-induced obesity is linked to elevated oxidative stress in both skeletal muscle and liver tissues, with skeletal muscle experiencing more pronounced oxidative stress than the liver [10].

Lipid peroxidation products, such as malondialdehyde (MDA), are significantly elevated in the skeletal muscle of obese rats, suggesting an increased level of lipid peroxidation [11]. Concurrently, the activity of certain key enzymes within the antioxidant oxidase system may be diminished, indicative of a weakened antioxidant defense mechanism [12]. Furthermore, the skeletal muscle of obese rats exhibits heightened inflammation, which is closely linked to oxidative stress. For instance, elevated levels of tumor necrosis factor α (TNF-α) and interleukin 6 (IL-6), among other inflammatory cytokines, not only contribute to the induction of oxidative stress but also result in muscle cell damage [13,14].

Intracellular NAD^+^ levels play a critical role in maintaining redox balance, and their close association with oxidative stress has prompted many scholars to investigate the relationship between nicotinamide adenine dinucleotide (NAD^+^) and high-fat diets. Studies have demonstrated that high-fat diets can significantly elevate hepatic NAD^+^ levels [15]. Exploring changes in NAD^+^ levels and consumption in obesity, as well as their enzymatic effects, aids in understanding high-fat diet-induced oxidative stress [16]. Enzymes involved in NAD^+^ consumption include the sirtuin protein family (Sirtuins), cluster of differentiation 38 (CD38), and poly (adenosine diphosphate-ribose) polymerase-1 (PARP-1). The increased activity of NAD^+^-consuming enzymes such as CD38 and PARP-1 leads to a reduction in NAD^+^ content, which directly affects the activity of Sirt proteins [16–19]. Sirt1 and Sirt3 are two subtypes of NAD^+^-dependent deacetylases that are highly expressed in skeletal muscle. Both Sirt1 and Sirt3 are regulated by peroxisome proliferator-activated receptor gamma coactivator 1-alpha (PGC1α), which promotes mitochondrial biogenesis and alleviates oxidative stress [20]. As a mitochondrial biogenesis transcription factor, PGC1α is a target of Sirt1 deacetylation [21]. Sirt1 protects muscle cells from oxidative stress by deacetylating and activating PGC1α [22], while PGC1α serves as an upstream regulator of Sirt3 and plays a crucial role in modulating oxidative stress [13]. Multiple studies indicate that Sirt3 is a novel target of PGC1α, contributing to the inhibition of oxidative stress and mitochondrial biogenesis [13,23]. Overexpression of Sirt3 reduces peroxide production [24].

Studies have shown that the antioxidant system is closely associated with the degree of obesity [25,26], however, little research has been done on how the oxidative stress state of the body changes during the obesity process. This study investigated the changes in oxidative stress, NAD^+^, and its consuming enzymes in the skeletal muscle of rats fed different high-fat diets at various time points. Furthermore, it examined the impact of the duration of high-fat diet exposure on oxidative stress, thereby providing a theoretical foundation for preventing oxidative stress during the progression of obesity in high-fat diet-induced populations.

## Materials and Methods

### Animal grouping and feeding programs

Thirty-two 6-week-old male SPF-grade Sprague-Dawley (SD) rats, aged six weeks and weighing 234.28±13.82 g, were purchased from Zhejiang Weitonglihua Experimental Technology Co., Ltd. The rats were housed in a standard specific pathogen-free (SPF) animal facility with controlled environmental conditions: room temperature of 24±4 °C, relative humidity of 50±10 %, and a 12-hour light-dark cycle (lights on from 8:00 to 20:00). They were group-housed in cages (4 rats per cage) with free access to water and standard adaptive feed for one week prior to the experiment. Subsequently, the rats were randomly assigned into four groups: control group (Group C, n=8), 4-week high-fat diet feeding group (H1 group, n=8), 8-week high-fat diet feeding group (H2 group, n=8), and 12-week high-fat diet feeding group (H3 group, n=8). The control group was fed a standard diet containing 4 % fat (Cat. No. P1101F-25, SLACOM, China), while the high-fat diet groups were provided with a diet containing 45 % fat (Cat. No. D12451, Research Diets, New Brunswick, USA). The detailed feeding scheme and grouping information are summarized in Table 1. During the 12-week experiment, the surplus grain from the previous day was cleared at a fixed time daily, an adequate amount of fresh feed was provided, and unified weighing was conducted promptly at 8:00 every weekend. All procedures used in this study were approved by The Nanjing Sports Institute Animals Experiment Ethics Committee and adhered to the international ethical standards [27].

### Blood sample collection and serum analysis

After 12 weeks, the rats were weighed and intraperitoneally anesthetized with a 20 % urethane solution (1 ml/100 g). Blood samples were collected from the abdominal aorta, and serum was obtained by centrifugation at 3000 rpm for 15 min at 4 °C. Serum samples were collected, and the concentrations of IL-6, TNF-α, and MCP-1 were assayed using the Rat Adipokine Magnetic Bead Panel (Cat. No. RADPNKMAG-80K, Merck KGaA, Darmstadt, Germany).

### Tissue collection and histological analysis

Gastrocnemius were collected and weighed. A portion of the gastrocnemius muscle tissue was fixed in 4 % paraformaldehyde solution for the preparation of paraffin sections to perform TUNEL staining analysis, whereas the remaining tissue was stored in liquid nitrogen for subsequent molecular biological assays. Gastrocnemius tissue was homogenized according to the protocol provided in the kit. The levels of NAD^+^ (Cat. No. CT-DS-5951, Shanghai Initial State), MDA (Cat. No. A003-1-2, Nanjing Jiancheng), SOD (Cat. No. A001-2-2, Nanjing Jiancheng), and GSH (Cat. No. A006-1-1, Nanjing Jiancheng) were quantified.

The middle portion of the left gastrocnemius muscle from rats was fixed in 4 % paraformaldehyde for 24 h, followed by rinsing with distilled water for 4 h post-fixation. Subsequently, dehydration was performed using a graded ethanol series (75 %, 90 %, 95 %, and 100 %) (Cat. No. 100092683, Sinopharm Group Chemical Reagents). The dehydrated tissue was cleared in xylene for 10 min and subsequently embedded in paraffin wax at melting points of 50–52 °C and 55–60 °C for 45 min and 1 h, respectively. After embedding, the paraffin-embedded gastrocnemius muscle was transferred to a paraffin embedding machine and cooled at −20 °C before being stored at room temperature or 4 °C. Sections were prepared using a manual microtome, cutting slices approximately 4 μm thick. These sections were then mounted on glass slides after being dried at 37 °C and used for subsequent experiments.

TUNEL staining was performed according to the manufacturer’s instructions provided in the TUNEL staining kit (Cat. No. C1090, Beyotime, China). Following staining, the sections were examined under an electron microscope at 200× magnification. For each sample, five non-overlapping fields of view were randomly selected for imaging. Image analysis software (Image J) was utilized to quantify the number of TUNEL-positive nuclei and total nuclei within each image. The TUNEL index was calculated as the ratio of TUNEL-positive nuclei to total nuclei, multiplied by 100.

### Tissue protein extraction, Western blotting and immunoprecipitation

An appropriate amount of gastrocnemius tissue was collected, and RIPA lysis buffer supplemented with protease inhibitors was added. The tissue was thoroughly homogenized using a grinder, and the supernatant was collected as the protein stock solution after centrifugation. Protein concentrations were determined according to the instructions provided in the BCA Protein Assay Kit (Cat. No. ZJ102). Subsequently, an equal volume of loading buffer (Cat. No. C508320-0001) was added to each sample. Proteins were denatured by heating at 95 °C for 5 min and then cooled to room temperature prior to SDS-PAGE electrophoresis. After electrophoresis, proteins were transferred onto a PVDF membrane to ensure complete transfer of proteins from the gel. The membrane was blocked with either 5 % bovine serum albumin (BSA) or 5 % skim milk (Cat. No. ST023) by incubating on a shaker at room temperature for 1.5 h. Following blocking, the membrane was washed three times with TBST buffer for 5 min each. Primary antibodies were prepared in 5 % skim milk or 5 % BSA according to the manufacturer’s instructions and diluted as indicated in Table 2. The membrane was incubated with the primary antibody overnight at 4 °C. After washing with TBST, the membrane was incubated with the corresponding secondary antibody (goat anti-rabbit/mouse IgG) for 1 h at room temperature. An appropriate volume of ultra-sensitive chemiluminescent substrate (mixed at a ratio of 1:1, liquid A:liquid B) was prepared and applied to the membrane for 30–60 s. The membrane was then visualized using the Bio-Rad ChemiDoc imaging system, and Image Lab software was used to analyze the grayscale intensity of the bands.

The tissue samples were thoroughly homogenized and lysed using a protein lysis buffer. The supernatant was collected, and the protein concentration was measured and adjusted to 6 μg/μl. Subsequently, 150 μl of each sample was extracted and pooled for immunoprecipitation. Cleaned magnetic beads were prepared on ice according to the manufacturer’s instructions provided in the immunoprecipitation kit (Cat. No. P2179S). Total PGC1α protein was precipitated, followed by Western blot analysis using an acetylated antibody to evaluate the expression level of acetylated PGC1α protein.

### Data analysis

All data were subjected to statistical analysis using IBM SPSS Statistics 25.0. The results are presented as mean ± standard error of the mean (SEM). The Shapiro-Wilk test was employed to confirm the normal distribution of the data. For comparisons between two groups, an independent samples *t*-test was conducted. One-way analysis of variance (ANOVA) was utilized for comparisons among three or more groups, followed by *post hoc* pairwise comparisons using the LSD method. Statistical significance was defined as P<0.05. Graphs were generated using GraphPad Prism 9.0 (San Diego, CA, USA) software.

## Results

### HFD increased the body weight and gastrocnemius muscle weight of rats

**Fig. 1:**
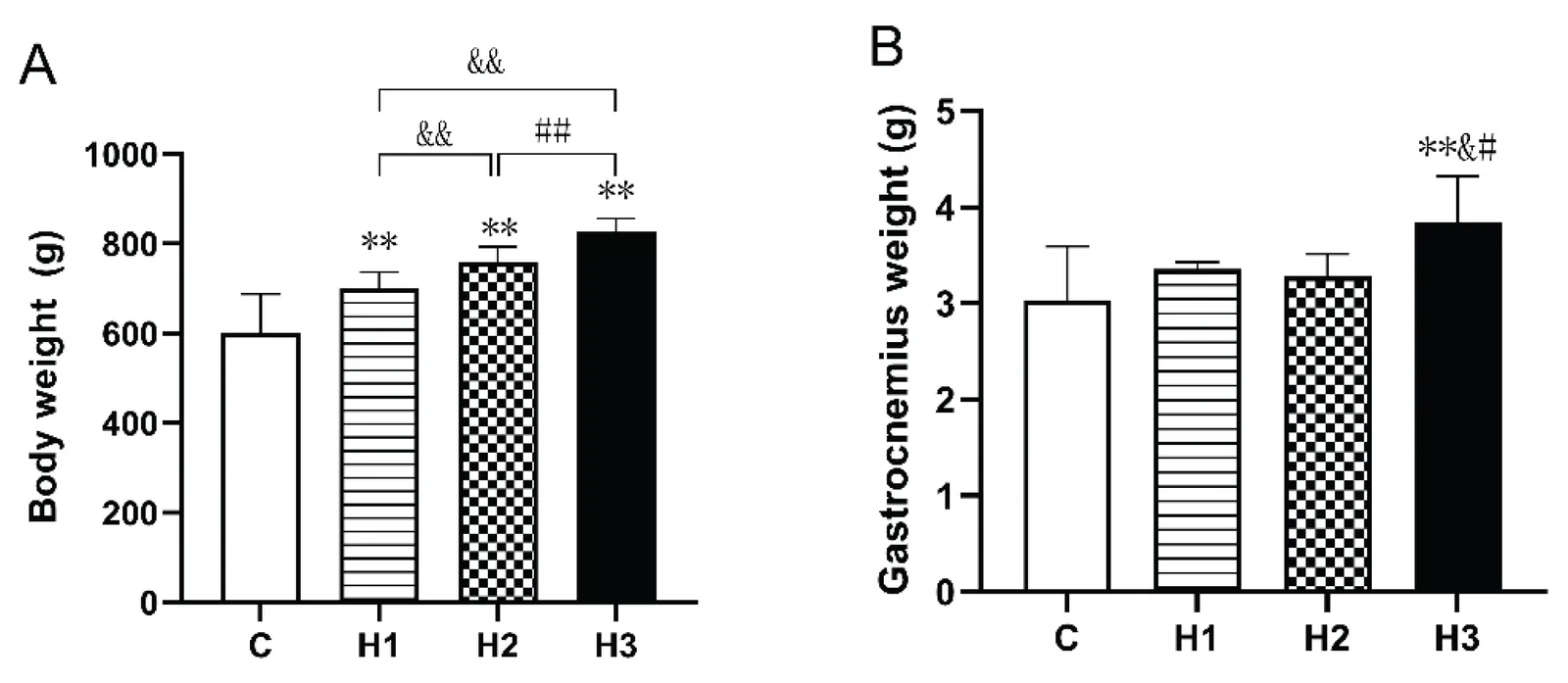
The body weight and gastrocnemius muscle weight of rats in each group. (**A**) Body weight. (**B**) Gastrocnemius weight. Values are expressed as mean ± SEM. Compared with group C, * P<0.05, ** P<0.01. Compared with group H1, ^&^ P<0.05, ^&&^ P<0.01. Compared with group H2, ^##^ P<0.01.

After 12 weeks of distinct dietary interventions, the body weights of rats in the H1, H2, and H3 groups were significantly higher than those in the C group (P<0.01, Fig. 1A). Additionally, the body weights in the H1, H2, and H3 groups exhibited an increasing trend, with significant differences observed among all groups (P<0.01, Fig. 1A). As depicted in Fig. 1B, compared to the C group, the gastrocnemius muscle weight of rats in the H3 group was markedly increased (P<0.01). Furthermore, when compared to the H1 group, the gastrocnemius muscle weight of rats in the H3 group showed a significant increase (P<0.05), and similarly, a significant increase was also observed when compared to the H2 group (P<0.05).

### Effect of different duration of high-fat diet on serum inflammatory factors and skeletal muscle oxidative stress in rats

**Fig. 2:**
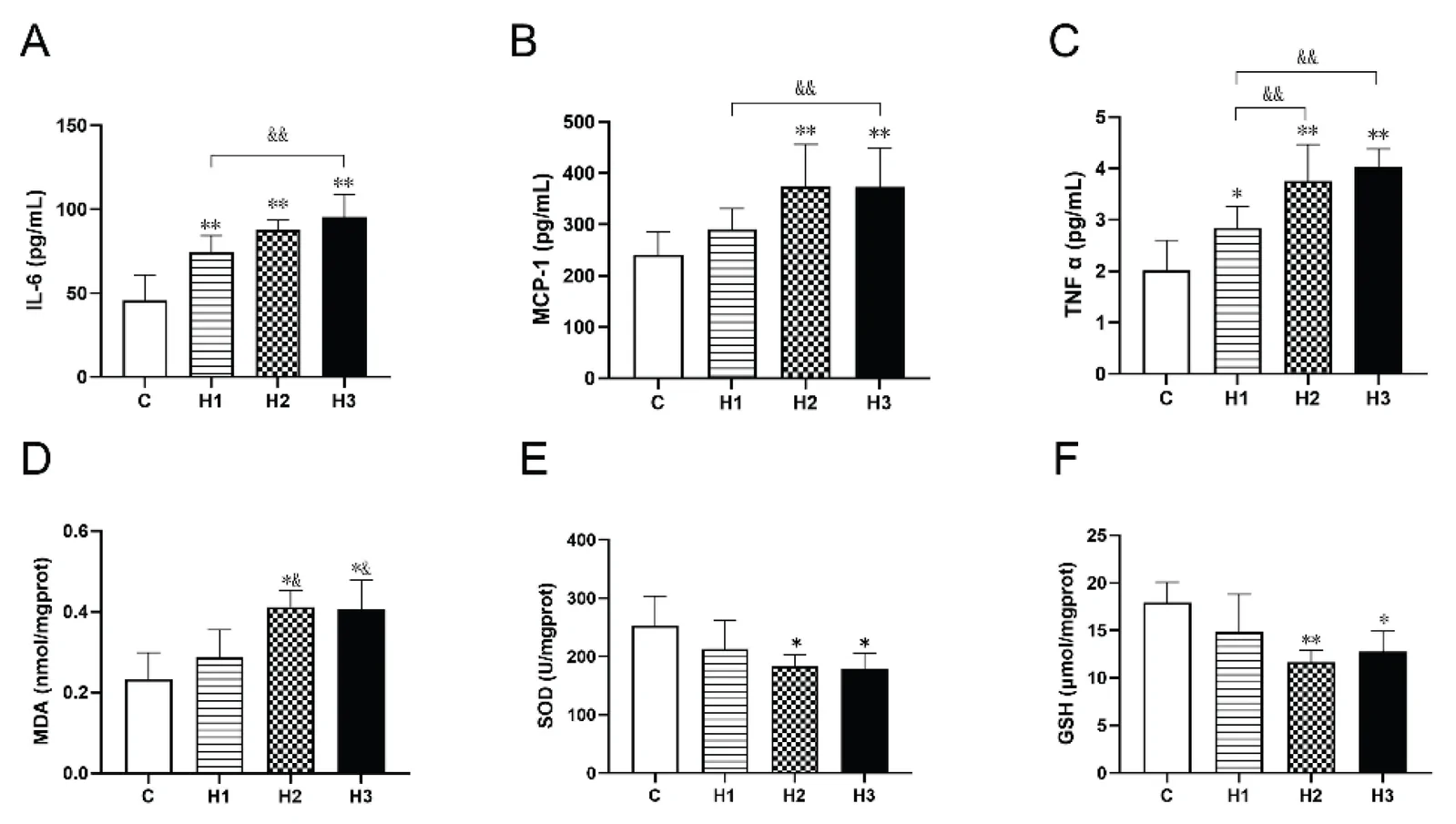
Expression of inflammatory factors in the blood of rats in each group, expression of oxidative and antioxidant markers in skeletal muscle. (**A**) interleukin-6 (IL-6). (**B**) monocyte chemotactic factor 1 (MCP-1). (**C**) tumor necrosis factor-α (TNF-α). (**D**) Malondial-dehyde (MDA). (**E**) superoxide dismutase (SOD). (**F**) glutathione (GSH). Values are expressed as mean ± SEM. Compared with group C, * P<0.05, ** P<0.01. Compared with group H1, ^&^ P<0.05, ^&&^ P<0.01.

As illustrated in Figure 2, compared with the difference was observed in MCP-1 levels (Fig. 2B), whereas TNF-α exhibited a significant increase (P<0.05, Fig. 2C). In the H2 and H3 groups, serum IL-6, MCP-1, and TNF-α levels were all significantly elevated (P<0.01, Fig. 2A, 2B, 2C). Compared with the H1 group, serum TNF-α levels in the H2 group were significantly increased (P<0.01, Fig. 2C), and all three inflammatory factors were significantly elevated in the H3 group (P<0.01, Fig. 2A, 2B, 2C). Relative to the control group (C), no significant differences were observed in MDA, SOD, and GSH levels in the skeletal muscle of rats in the H1 group (P>0.05). However, in the H2 group, MDA levels in skeletal muscle were significantly increased (P<0.05; Fig. 2D), while SOD levels were significantly reduced (P<0.05; Fig. 2E), and GSH levels were also significantly decreased (P<0.01; Fig. 2F). In the H3 group, MDA levels in skeletal muscle were significantly increased (P<0.05; Fig. 2D), and both SOD and GSH levels were significantly reduced (P<0.05).

### Effect of different duration of high-fat diet on apoptosis in rat gastrocnemius muscle cells

**Fig. 3:**
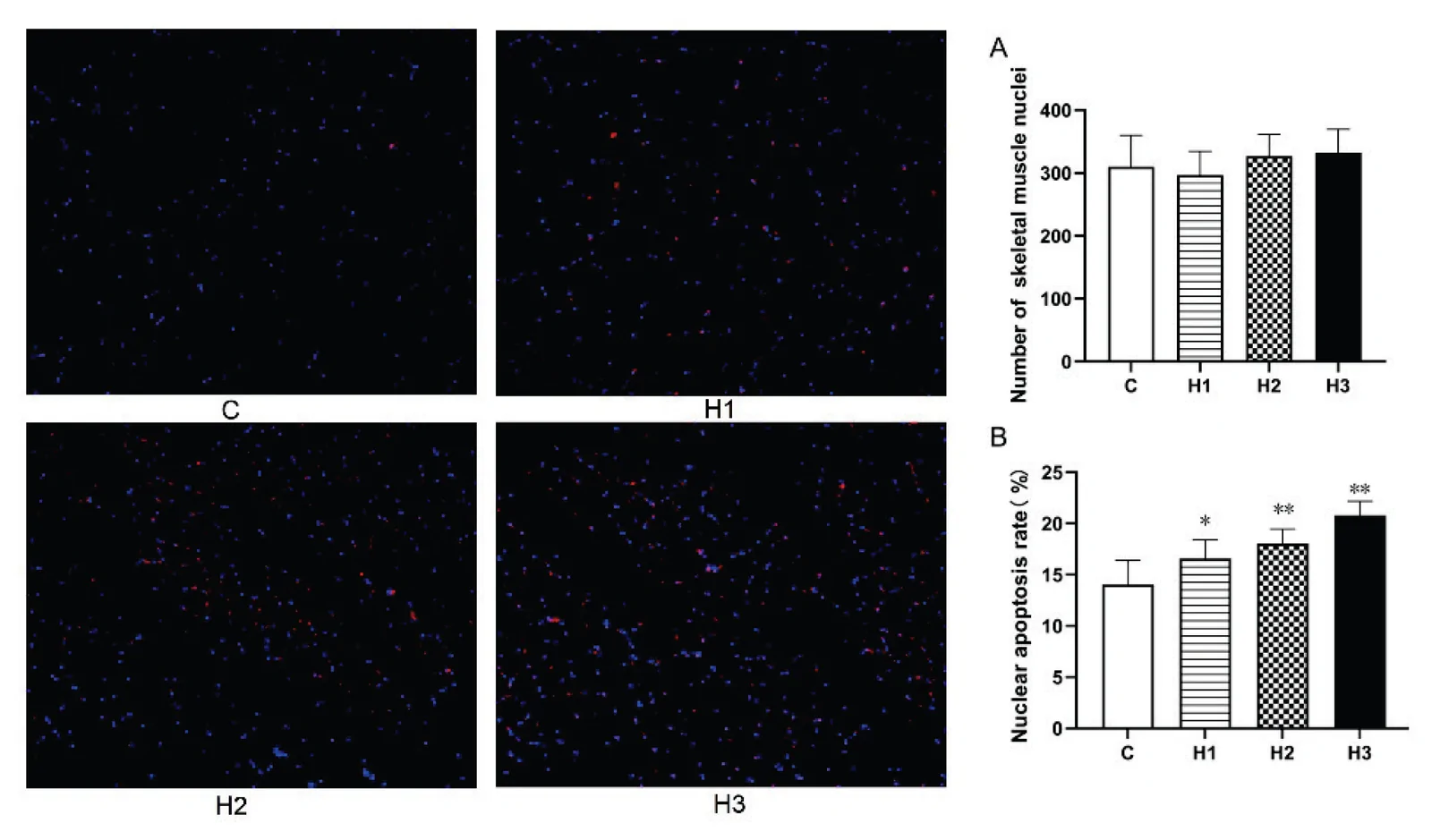
TUNEL staining and myocyte apoptosis of gastro-cnemius muscle in each group. TUNEL staining of gastrocnemius muscle in groups C, H1, H2, H3. (**A**) total number of nuclei of myoblasts in each group. (**B**) apoptosis rate of rat myoblasts in each group. Values are expressed as mean ± SEM. compared with group C, * P<0.05, ** P<0.01; Image magnification: 200×. Scale bar: 50 μm.

As illustrated in Figure 3, when compared with Group C, no statistically significant difference was observed in the total number of muscle nuclei in the gastrocnemius of rats in Groups H1, H2, and H3 (P>0.05, Fig. 3A). The nuclear apoptosis rate in Group H1 was significantly elevated (P<0.05, Fig. 3B), whereas those in Groups H2 and H3 were extremely significantly increased (P<0.01, Fig. 3B).

### Effects of varying durations of High-Fat Diet on NAD^+^/Sirtuins/PGC1α pathway in rat gastrocnemius muscle

**Fig. 4:**
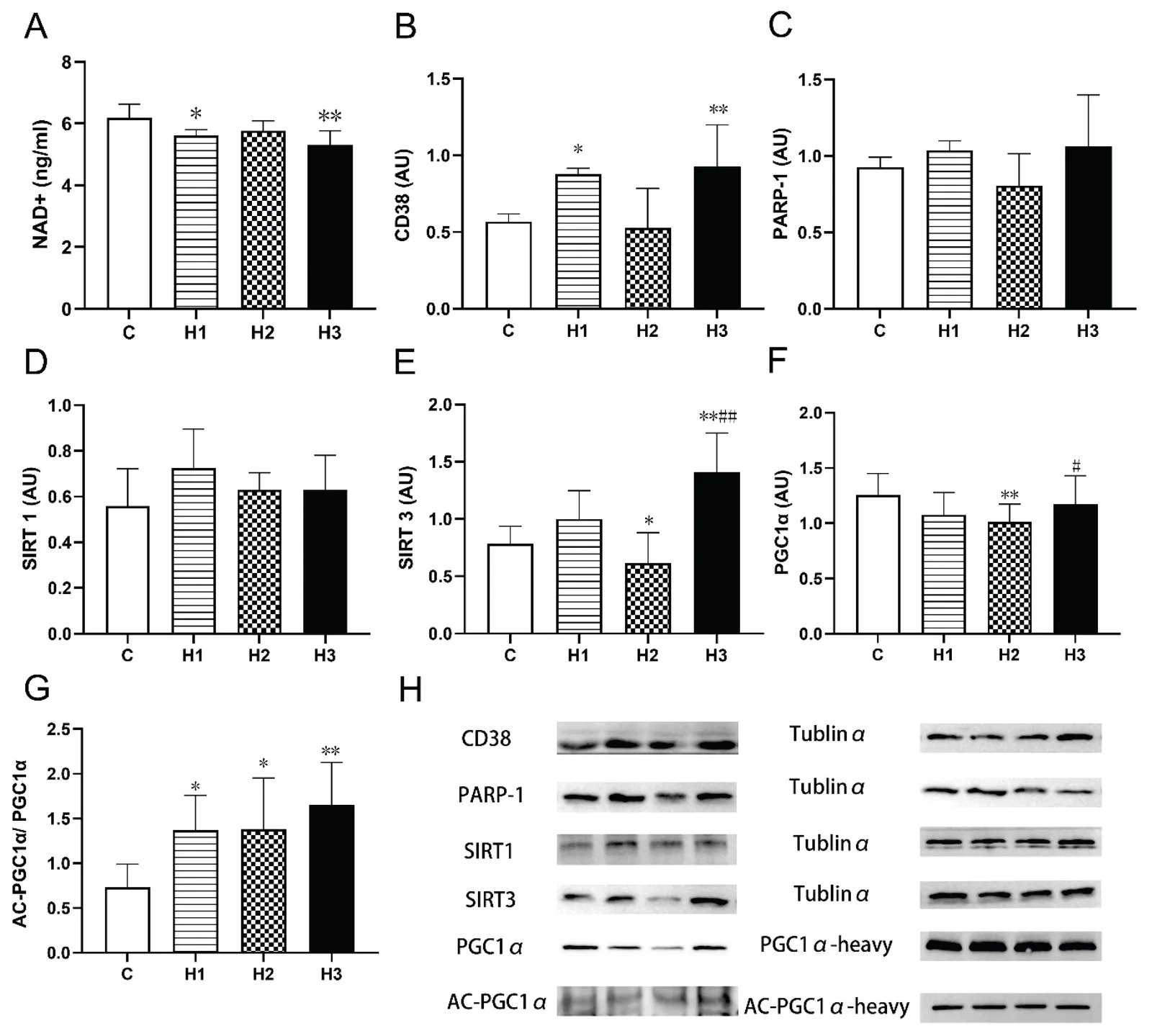
Expression of NAD^+^/Sirtuins/PGC1α regulators of oxidative stress in rats in each group. (**A**) NAD^+^ content levels (**B–G**) Western blot analysis of CD38, PARP-1, SIRT1, SIRT3, PGC1α, AC-PGC1α/PGC1α. (**H**) Western blot images. Values are expressed as mean ± SEM. Compared with group C, * P<0.05, ** P<0.01. Compared with group H2, ^#^ P<0.05, ^##^ P<0.01. AU, arbitrary unit.

As illustrated in Figure 4, compared with group C, the NAD^+^ content in group H1 was significantly reduced (P<0.05), while the expression of CD38 protein was markedly elevated (P<0.05). No significant differences were observed in the expression levels of PARP-1, SIRT-1, SIRT-3, and PGC1α proteins in group H1 (P>0.05). However, the proportion of acetylated PGC1α protein was significantly increased (P<0.05). In group H2, there were no significant changes in NAD^+^ content or the expressions of CD38, PARP-1, and SIRT-1 proteins (P>0.05). Conversely, the expression of SIRT-3 protein was significantly decreased (P<0.05), and the expression of PGC1α protein was significantly reduced (P<0.01). Additionally, the proportion of acetylated PGC1α protein was significantly increased (P<0.05). In group H3, the NAD^+^ content was significantly diminished (P<0.05), and the expression of CD38 protein was significantly upregulated (P<0.01). The expression levels of PARP-1 and SIRT-1 proteins remained unchanged (P>0.05), whereas the expression of SIRT-3 protein was significantly enhanced (P<0.01). No significant differences were detected in the expression of PGC1α protein (P>0.05), but the proportion of acetylated PGC1α protein was significantly increased (P<0.01). Compared with group H2, the expression levels of SIRT3 and PGC1α proteins in group H3 were significantly higher (P<0.05).

## Discussion

During the 12-week modeling period, rats were fed a high-fat diet for 4, 8, and 12 weeks, respectively. Their body weights increased by 10 %, 20 %, and 30 %, respectively. Through the detection of inflammatory factors, oxidative stress, and nuclear apoptosis, it was observed that the inflammatory response and cell apoptosis in the three groups of rats fed with high-fat diets were exacerbated as obesity levels increased. Significant differences were noted in the oxidative stress levels in the skeletal muscles of rats subjected to varying durations of high-fat diets. After 4 weeks of high-fat diet administration, only the apoptosis rate of the gastrocnemius muscle increased; however, with prolonged exposure to the high-fat diet, oxidative stress levels in skeletal muscles significantly increased. During the 8- and 12-week periods of high-fat diet feeding, the content of malondialdehyde (MDA), a lipid peroxidation product, in skeletal muscles markedly increased, reflecting intensified cellular lipid peroxidation. Simultaneously, the levels of antioxidant enzymes superoxide dismutase (SOD) and glutathione (GSH) significantly decreased, indicating severe inhibition of the antioxidant defense system. Additionally, the intensification of inflammatory responses, such as tumor necrosis factor-alpha (TNF-α) and interleukin-6 (IL-6), along with elevated levels of inflammatory cytokines, promoted the exacerbation of oxidative stress. These findings align with previous studies on obesity models [12]. In conclusion, a 4-week high-fat diet initially induces changes in the body’s inflammatory response, while 8- and 12-week high-fat diets lead to increased peroxidation reactions and reduced antioxidant capacity in tissues. The inflammatory response and oxidative stress are further enhanced with prolonged high-fat diet exposure, underscoring the differential effects of varying high-fat diet durations on systemic oxidative stress.

Following four weeks of a high-fat diet, rat body weight increased by 10 %, serum inflammatory factors TNF-α and IL-6 were markedly elevated, the levels of MDA, SOD, and GSH in rat skeletal muscle remained unchanged, while skeletal muscle NAD^+^ levels were significantly reduced, which correlated with an increase in the consumption enzyme CD38, while PARP-1 levels remained unchanged. These findings suggest that the upregulation of the NAD^+^-consuming enzyme CD38 led to decreased NAD^+^ levels, subsequently reducing Sirt1 deacetylation activity and increasing the acetylation ratio of PGC1α. However, four weeks of a high-fat diet did not significantly affect PGC1α protein levels or its downstream target Sirt3, indicating that this duration of a high-fat diet initiates systemic inflammation and disrupts the NAD^+^/Sirt1/PGC1α without inducing significant oxidative stress.

Following an 8-week high-fat diet intervention, compared to the control group, serum inflammatory factors (TNF-α, IL-6, and MCP-1) were significantly elevated in rats. Additionally, there was no significant difference in MDA, a significant decrease in SOD, and a highly significant decrease in GSH in rat skeletal muscle. Notably, NAD^+^ content did not exhibit significant changes. Similarly, the contents of NAD^+^-consuming enzymes CD38 and PARP-1 in skeletal muscle remained unchanged, as did the Sirt1 protein, which is highly dependent on NAD^+^ levels. However, the expression of PGC1α protein was significantly reduced, whereas the proportion of acetylated PGC1α increased significantly. This suggests that the increase in acetylated PGC1α may result from the marked reduction in PGC1α levels. With the decline in PGC1α, Sirt3 expression also decreased significantly, indicating that the exacerbation of oxidative stress induced by the 8-week high-fat diet might be attributed to the downregulation of the PGC1α/Sirt3. Furthermore, the increased proportion of acetylated PGC1α appears to contribute to enhanced oxidative stress. Previous studies have demonstrated that the effects of a high-fat diet on Sirt1 are primarily reflected in deacetylation activity [16]. After 8 weeks of high-fat diet exposure, neither NAD^+^ nor Sirt1 exhibited significant changes; however, the proportion of acetylated PGC1α increased, suggesting that an 8-week high-fat diet may exacerbate oxidative stress through the regulation of PGC1α and Sirt3.

After 12 weeks of high-fat diet feeding, the observed trends were consistent with those after 4 weeks of high-fat diet feeding but exhibited more pronounced effects. Specifically, malondialdehyde (MDA) levels were significantly increased, while superoxide dismutase (SOD) and glutathione (GSH) levels were significantly decreased. Additionally, NAD^+^ content was markedly reduced, accompanied by a significant increase in the consumption enzyme CD38, leading to a decrease in NAD^+^ levels. This subsequently affected the deacetylation activity of Sirt1 and resulted in a significant increase in the acetylation ratio of PGC1α. Notably, the total PGC1α content did not change significantly compared to the control group, thus excluding the possibility that the decrease in total PGC1α influenced the increase in the acetylated PGC1α proportion. In conclusion, the 12-week high-fat diet led to a further decline in the activity of the NAD^+^/Sirt1/PGC1α, enhancing oxidative stress. It is worth noting that Sirt3 expression was significantly upregulated. After 8 weeks of high-fat diet feeding, rat body weight increased by 20 %, and skeletal muscle PGC1α and Sirt3 protein levels were significantly reduced, with concomitant enhancement of oxidative stress. Following 12 weeks of high-fat diet feeding, PGC1α levels slightly recovered, Sirt3 expression increased, yet oxidative stress remained unchanged. These findings suggest that after 12 weeks of high-fat diet feeding, rat body weight increases by over 30 %. At this stage, fat cells accumulate and deposit within muscles, forming intermuscular fat [28], which induces inflammation and promotes oxidative stress [29]. The entire organism enters a chronic inflammatory state [30]. Studies have demonstrated that when fat accumulation reaches a critical threshold, visceral and subcutaneous fat expands, causing fat cells to infiltrate between muscle fibers or surrounding muscles. The increased influx of fatty acids and inflammatory molecules, particularly from visceral adipose tissue, induces muscle inflammation and negatively impacts muscle cell metabolism, contributing to insulin resistance [30]. In this study, after 12 weeks of high-fat diet feeding, inflammatory factors IL-6, MCP-1, and TNF-α in rat skeletal muscle were significantly elevated, corroborating these findings. During the insulin resistance phase, enhanced oxidative stress coincides with higher levels of AMP-activated protein kinase (AMPK) activation, resulting in significant upregulation of PGC1α protein levels [30]. Consequently, the upregulation of PGC1α at this stage leads to increased Sirt3 expression. This could explain the coexistence of increased skeletal muscle oxidative stress alongside elevated PGC1α and Sirt3 expression after 12 weeks of high-fat diet feeding. However, despite the increase in Sirt3, it failed to reverse oxidative stress due to the severe inflammatory response induced by adipose tissue infiltration, which concurrently affected metabolism and intensified oxidative stress. Our previous studies on adipose tissue provide strong support for this perspective [33].

In summary, after 12 weeks of high-fat diet feeding, rats exhibit excessive obesity, which results in a significant reduction in the NAD^+^/Sirt1/PGC1α within skeletal muscle. Simultaneously, the infiltration of adipose tissue-derived inflammatory cells into skeletal muscle leads to elevated levels of inflammatory cytokines and exacerbates oxidative stress. Even though PGC1α/Sirt3 expression is increased, it is insufficient to counteract the severe oxidative stress.

## Shortcomings and Prospects

This study systematically investigated the effects of high-fat feeding at different durations on oxidative stress in rat skeletal muscle. It was found that both 8-week and 12-week high-fat feeding regimens exacerbated oxidative stress in rat skeletal muscle, albeit potentially through distinct mechanisms. However, several limitations were identified: firstly, the impact of oxidative stress on mitochondrial function was not adequately addressed, as reactive oxygen species (ROS) and mitochondrial bioenergetics indices were not promptly assessed during sampling; secondly, the analysis of Sirtuins proteins was insufficient, leaving the roles of PGC1α and Sirt3 in modulating oxidative stress unexplored. Future research could delve deeper into the impact of high-fat diet duration on mitochondrial function and the Sirtuins antioxidant system.

## Conclusions

**Fig. 5:**
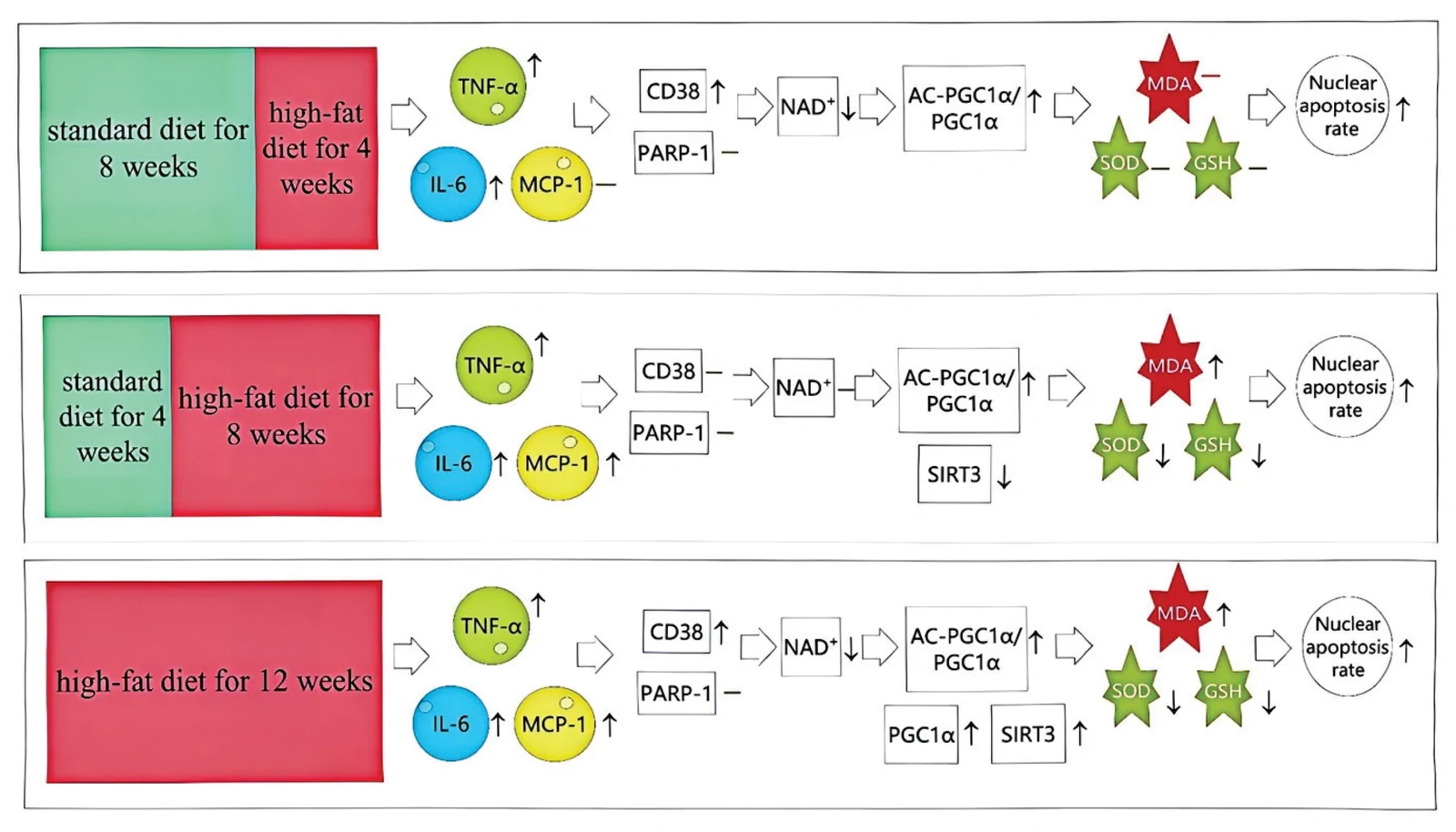
Summary diagram.

There were differences in the effects of varying durations of high-fat diet feeding on oxidative stress in rat skeletal muscle. With prolonged high-fat feeding, systemic inflammation progressively intensified and the extent of oxidative stress increased. After 4 weeks of high-fat feeding, rats exhibited elevated inflammatory markers and a significant reduction in NAD^+^ levels, although no evident oxidative stress was detected. Following 8 weeks of high-fat feeding, rats demonstrated elevated lipid peroxidation, reduced antioxidant capacity, decreased PGC1α/Sirt3 expression in skeletal muscle, and heightened oxidative stress. After 12 weeks of high-fat feeding, NAD^+^/Sirt1 levels further declined, exacerbating oxidative stress in skeletal muscle. Despite a significant increase in acetylated PGC1α/Sirt3, this did not mitigate the worsening oxidative stress condition (Fig. 5).

## Figures and Tables

**Table 1:** Animal feeding programs and groupings.

Group	Feeding program
*C (control group)*	Feeding on standard diet for 12 weeks
*H1 (high-fat diet for 4 weeks)*	Feeding on standard diet for 8 weeks and high-fat diet for 4 weeks
*H2 (high-fat diet for 8 weeks)*	Feeding on standard diet for 4 weeks and high-fat diet for 8 weeks
*H3 (high-fat diet for 12 weeks)*	Feeding on high-fat diet for 12 weeks

**Table 2:** Antibody sources and dilution ratios.

Antibodies	Dilution	Number	Company
*CD38*	1:1000	BS80063	Bioworld
*PARP-1*	1:750	BS7047	Bioworld
*SIRT1*	1:750	MB11597	Bioworld
*SIRT3*	1:500	BS7772	Bioworld
*PGC1α*	1:500	66369-1-Ig	PTG
*Ac-lysine*	1:500	sc-542937	Santa cruz
*m-IgG1 BP-HRP*	1:5000	sc-542937	Santa cruz
*Tubulin α*	1:10000	BS1699	Bioworld
